# Egg White Protein Feeding Facilitates Skeletal Muscle Gain in Young Rats with/without Clenbuterol Treatment

**DOI:** 10.3390/nu13062042

**Published:** 2021-06-15

**Authors:** Keiichi Koshinaka, Asuka Honda, Rihei Iizumi, Yuto Miyazawa, Kentaro Kawanaka, Akiko Sato

**Affiliations:** 1 Department of Health and Sports, Niigata University of Health and Welfare, 1398 Shimami-cho, Kita-ku, Niigata 950-3198, Japan; wtm19009@nuhw.ac.jp (A.H.); wta16014@nuhw.ac.jp (R.I.); wta17191@nuhw.ac.jp (Y.M.); akiko-sato@nuhw.ac.jp (A.S.); 2 Faculty of Sports and Health Science, Fukuoka University, 8-19-1 Nanakuma, Jonan-ku, Fukuoka 814-0180, Japan; kawanaka@fukuoka-u.ac.jp

**Keywords:** egg white protein, casein protein, whey protein, muscle gain, clenbuterol, arginine, mTOR

## Abstract

Based on the Digestible Indispensable Amino Acid Score (DIAAS), egg white protein (EGG) has an excellent score, comparable to that of whey protein but with a lower amount of leucine. We examined the effect of EGG feeding on rat skeletal muscle gain in comparison to that of two common animal-derived protein sources: casein (CAS) and whey (WHE). To explore the full potential of EGG, this was examined in clenbuterol-treated young rats. Furthermore, we focused on leucine-associated anabolic signaling in response to EGG after single-dose ingestion and chronic ingestion, as well as clenbuterol treatment. Because EGG is an arginine-rich protein source, a portion of the experiment was repeated with diets containing equal amounts of arginine. We demonstrated that EGG feeding accelerates skeletal muscle gain under anabolism-dominant conditions more efficiently than CAS and WHE and this stronger effect with EGG is not dependent on the arginine-rich composition of the protein source. We also demonstrated that the plausible mechanism of the stronger muscle-gain effect with EGG is not detectable in the mechanistic target of rapamycin (mTOR) or insulin signaling under our experimental conditions. We conclude that EGG may have a superior efficiency in muscle gain compared to other common animal-based proteins.

## 1. Introduction

Skeletal muscle mass is moderated by the balance between the rate of protein synthesis and protein breakdown, both of which are highly influenced by dietary protein intake [1]. Different types of protein sources cause the efficiency of muscle anabolism to vary, along with the amount of protein consumed. Therefore, the daily intake of high-quality protein through diet or supplementation has been recognized as a possible strategy for maximizing nutritional and training-induced anabolic effects on the skeletal muscle of athletes as well as older subjects experiencing sarcopenia [2,3]. Protein quality is mainly defined by the amount of essential amino acids and their bioavailability and digestibility, which are scored by various calculations [4]. Based on the Digestible Indispensable Amino Acid Score (DIAAS), animal-derived protein sources generally have higher scores than those of plant-derived proteins [5]. In accordance with this concept, several researchers have focused on the anabolic effect of animal-derived whey protein (WHE), the most popular protein source used in supplements on the consumer market. In comparison to soy protein, previous studies have demonstrated that single-dose (acute) WHE ingestion produced more muscle protein synthesis [6,7], and chronic WHE ingestion with resistance training led to more muscle gain [8]. This difference in anabolic effects among protein sources is at least partially attributed to different amounts of branched-chain amino acids (BCAA), particularly leucine, which is well characterized as a critical and powerful stimulant for inducing anabolic effects on skeletal muscle [9]. Leucine can augment muscle protein synthesis independently of all other amino acids through activation of the mechanistic target of rapamycin complex 1 (mTORC1) signaling which is thought to be a primary pathway for muscle protein synthesis [9,10,11]. In parallel, the activation of the protein kinase B (Akt) pathway in insulin signaling also results in the activation of the mTORC1 pathway with simultaneous inhibition of protein breakdown by Forkhead box-containing protein O1 (FOXO1) [9]. The importance of sufficient dietary leucine to facilitate muscle protein synthesis is supported by previous studies showing that suboptimal ingestion of low amounts of protein stimulated muscle protein synthesis compared to the levels achieved with high amounts of protein when leucine was added [12,13].

WHE is leucine-rich (8.6 g/100 g protein) compared to typical plant-derived soy protein (5 g/100 g protein). Interestingly another animal-derived protein, egg white protein (EGG), contains lower levels of leucine (3.6 g/100 g protein) [14] but has an excellent score of 1.45 according to the Digestible Indispensable Amino Acid Score (DIAAS) formula and is comparable to whey protein (1.45 in DIAAS) [15]. Indeed, a previous study provided evidence that EGG supplementation in young adult males for 5 weeks, along with exercise training, effectively facilitated muscle strength adaptation with a large gain in lean body mass and cross-sectional area of skeletal muscle [16]. This suggests EGG is a useful and effective protein source for obtaining more muscle, but this anabolic effect has not been compared with that of other sources of protein supplementation [16]. While several studies did compare CAS versus WHE, or WHE versus plant-derived protein sources, there is scant information regarding the effectiveness of EGG on muscle anabolism compared to other excellent animal-derived protein sources [2]. In comparison to casein (CAS), EGG feeding reduced visceral fat mass in insulin-resistant animals [17,18,19]. A recent study conducted by Matsuoka et al. [20] demonstrated in an animal study that EGG ingestion resulted in greater body weight gain, food efficiency (body weight gain/food consumption), and protein efficiency (body weight gain/protein intake) compared to WHE. Although these authors did not discuss skeletal muscle mass, this result has given rise to the hypothesis that EGG might have a superior anabolic effect on skeletal muscle compared to other animal-derived proteins such as CAS and WHE, nevertheless with a lower amount of leucine.

Therefore, in the present study, we examined the effect of EGG feeding on rat skeletal muscle gain in comparison to that of the common animal-derived protein sources CAS and WHE. To emphasize the potential of EGG, the effect was examined in clenbuterol-treated young rats, which is considered as the condition of being highly anabolism-dominant. We hypothesize that EGG feeding would result in greater muscle gain. Furthermore, we focused on the activation level of leucine-associated anabolic signaling in response to EGG after single-dose ingestion and after chronic ingestion, along with the interaction of clenbuterol treatment. Because EGG is an arginine-rich protein source and arginine functions in an anabolic hormone secretion promoter [21,22], a portion of the experiment was repeated with diets containing equal amounts of arginine.

## 2. Materials and Methods

### 2.1. Animals

This research was approved by the Animal Studies Committee of Niigata University of Health and Welfare (29006-02103, 29016-30005, 02009, 29016). Male Wistar rats (4 weeks old) were obtained from CLEA Japan (Tokyo, Japan). Rats were housed individually at constant room temperature (23 ± 1 °C) in a 12-h light (06:00−18:00 h) and 12-h dark cycle and were provided standard laboratory chow (MF: Oriental Yeast, Tokyo, Japan) and water ad libitum until the experiment commenced. After an acclimatization period of one week, all rats were used in the experiment.

### 2.2. Experimental Diets

The exact composition of each protein source is shown in Table 1. According to these amounts, the protein concentration in Diet 1 was set as 20 g in 100 g diet (original diet). All materials, with the exception of protein sources, were obtained from Oriental Yeast (Tokyo, Japan). CAS (Calcium Caseinate A, DMV, Netherlands), EGG (EAP-sport PLUS: SCETI, K.K., Japan), and WHE (Wheyco W80, Nippon Shinyaku, Japan) were obtained from SCETI, K.K., Japan. The amino acid composition of Diet 1 is shown in Table 2. To make the arginine concentration in the CAS and WHE diets equal to that of the EGG diets, arginine was added to both the CAS diet (460 mg/100 g) and WHE diet (632 mg/100 g), with the counter-addition of proline to the CAS diet (172 mg/100 g) and EGG diet (632 mg/100 g). The final amino acid composition in these arginine-modified diets is shown as Diet 2 in Table 2.

### 2.3. Experiment I: Acute Effect of EGG Feeding on Muscle Signal Transduction (on Day 0) under Normal Conditions (without Clenbuterol Treatment)

**Figure 1:**
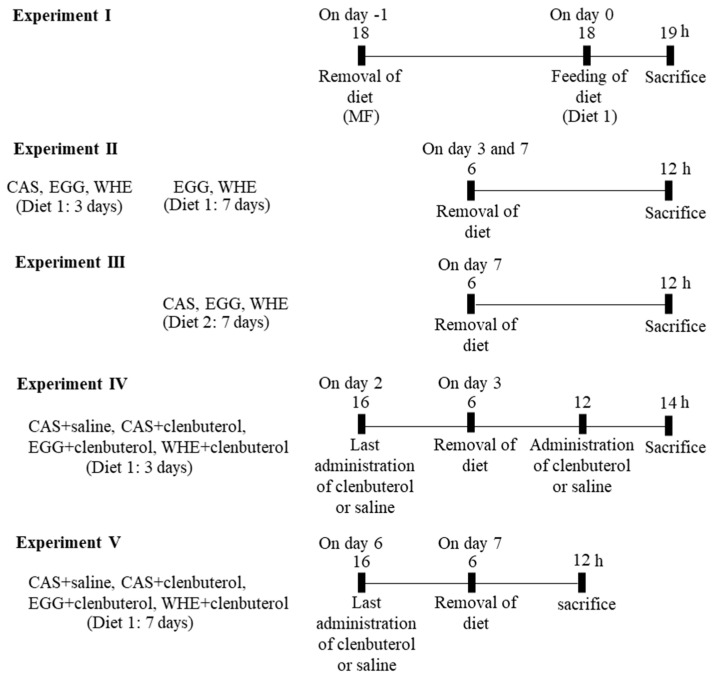
Experimental design.

Rats were randomly assigned to either a fasting, CAS, EGG, or WHE group (Figure 1). After 24 h of fasting, rats were allowed to feed with Diet 1 containing the assigned protein, while rats in the fasting group were maintained in the fasting state. One hour after feeding, blood was taken, and the plantaris muscles were clamp-frozen in liquid nitrogen for western blot analysis.

### 2.4. Experiment II: Chronic (7-Day) Effect of EGG Feeding under Normal Conditions (without Clenbuterol Treatment)

Rats were randomly assigned to one of the CAS, EGG, or WHE groups, and were maintained on Diet 1 and clenbuterol treatments for 3 or 7 days, as described above, except for saline or clenbuterol administration (Figure 1). On day 7, after 6 h of fasting, fat pads and skeletal muscles were dissected out under anesthesia and weighed. On day 3, some rats were sacrificed after 6 h of fasting, and plantaris muscles were clamp-frozen in liquid nitrogen for western blot analysis.

### 2.5. Experiment III: Chronic Effect of EGG Feeding on Arginine Modification (without Clenbuterol Treatment)

Rats were randomly assigned to either a CAS, EGG, or WHE group, and were fed the arginine-modified Diet 2 containing the respective protein source (Figure 1). They were fed ad libitum for 7 days. On day 7, after the 6-h fasting, fat pads and skeletal muscles were dissected out under anesthesia and weighed.

### 2.6. Experiment IV: Acute Effect of Clenbuterol on Muscle Signal Transduction on Day 3

Rats were randomly assigned to one of the CAS+saline, CAS+clenbuterol, EGG+clenbuterol, or WHE+clenbuterol groups, and were maintained for 2 days on Diet 1 for feeding and clenbuterol treatments as above (Figure 1). On day 3, after 6 h of fasting and 20 h after the last clenbuterol administration, the rats received intraperitoneal injections of clenbuterol (1 mg/kg body weight) or saline alone. The rats were maintained in a fasting state under resting conditions for 2 h. Following this, the plantaris muscles were dissected out under anesthesia and clamp-frozen in liquid nitrogen for western blot analysis.

### 2.7. Experiment V: Chronic (7-Day) Effect of EGG Feeding with Clenbuterol

Rats were randomly assigned to one of the CAS+saline, CAS+clenbuterol, EGG+clenbuterol, or WHE+clenbuterol groups, and were fed Diet 1 of the respective protein source ad libitum for 7 days (Figure 1). During this period, rats received once-daily intraperitoneal administration of either clenbuterol (1 mg/kg body weight: Toronto Research Chemicals, North York, Canada) dissolved in saline (2 mL/kg body weight) or saline alone, with daily measurement of food intake. On day 6, the last intraperitoneal administration was at 16:00. The next morning (at 06:00), the food was removed, and rats were maintained in the fasting state for 6 h. Following this, fat pads (epididymal and retroperitoneal) and skeletal muscles (soleus and plantaris) were dissected out under anesthesia and weighed. The plantaris muscles were clamp-frozen in liquid nitrogen for western blot analysis.

### 2.8. Western Blot Analysis

Plantaris muscles were homogenized in ice-cold buffer containing 50 mM HEPES (pH 7.4), 150 mM NaCl, 10% glycerol, 1% Triton X-100, 1.5 mM MgCl_2_, 1 mM EDTA, 10 mM Na_4_P_2_O_7_, 100 mM NaF, 2 mM Na_3_VO_4_, 2 mM PMSF, aprotinin (10 μg/mL), and leupeptin (10 μg/mL). The homogenates were rotated end-over-end at 4 °C for 60 min and then centrifuged at 10,000× *g* for 10 min at 4 °C. Aliquots of supernatants were used for immunoblot analysis. Briefly, supernatants were electrophoretically separated by SDS-PAGE and transferred to PVDF membranes. The membranes were incubated overnight at 4 °C with primary antibodies, followed by incubation for 60 min with appropriate HRP-conjugated second antibodies. Primary antibodies against mTOR, phospho-mTOR (Ser2448), Akt, phospho-Akt (Thr308), S6, and phospho-S6 (Ser235/236), cAMP response element-binding protein (CREB), and phospho-CREB (Ser133) were obtained from Cell Signaling Technology (Beverly, MA). Immunoreactive bands were visualized using enhanced chemiluminescence reagent (GE Healthcare Japan, Hino, Japan), and quantified using NIH Image software.

### 2.9. Blood Parameters

Blood glucose levels were measured using the product Glutest Every (Sanwa Kagaku, Nagoya, Japan). Enzyme immunoassay kits were used for measuring insulin (Morinaga Institute of Biological Science, Yokohama, Japan), growth hormone (SPI-BIO, Montigny le Bretonneux, France), and IGF-1 ((R&D Systems, Minneapolis, MN, USA).

### 2.10. Amino Acid and Protein Compositions

The measurements of protein content in each protein source and the composition of amino acids in each diet were conducted by Sunatec, food analysis technology center, Yokkaichi, Japan.

### 2.11. Statistics

Values are expressed as means ± SE. Differences among multiple groups were determined using a one-way analysis of variance (ANOVA) followed by the Tukey–Kramer test. When two mean values were compared, analysis was performed using an unpaired *t*-test. A value of *p* < 0.05 was considered significant.

## 3. Results

### 3.1. Experiment I

**Figure 2:**
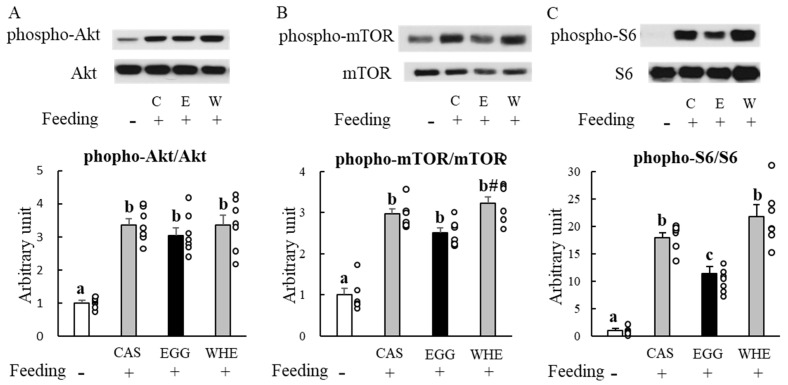
Acute effect of each protein feeding on muscle signal transduction (Day 0). Rats were assigned to either a casein, egg white, or whey protein diet group. After 24-h fasting, rats were allowed a feeding diet containing assigned protein. One hour after the feeding, plantaris muscles were dissected out for western blot analysis of Akt (**A**), mTOR (**B**), or S6 (**C**). The dots beside bars indicate individual value. Values are means ± SE (*n* = 7). CAS (C): casein protein, EGG (E): egg white protein, WHE (W): whey protein. Different superscript represents statistical differences among groups. #: *p* < 0.05 vs. EGG group.

**Figure 3:**
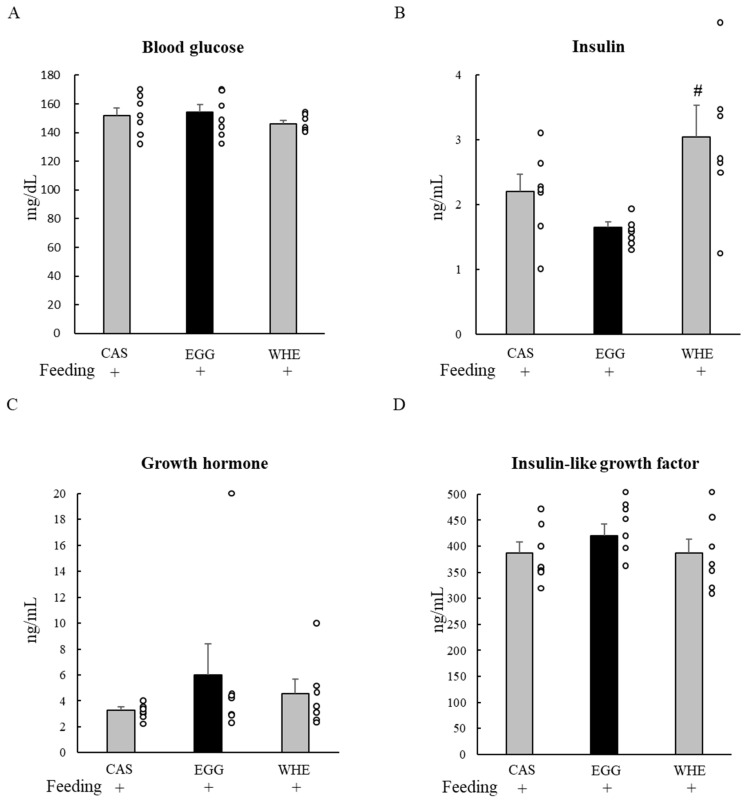
Acute effect of each protein feeding on blood parameters (Day 0). Rats were assigned to either a casein, egg white, or whey protein diet group. After 24-h fasting, rats were allowed a feeding diet containing assigned protein. One hour after the feeding, bloods were taken, and then were analyzed for measuring blood glucose (**A**), insulin (**B**), growth hormone (**C**), or insulin-like growth factor 1 (**D**). The dots beside bars indicate individual value. Values are means ± SE (*n* = 7). CAS: casein protein, EGG: egg white protein, WHE: whey protein. Different superscript represents statistical differences among groups. #: *p* < 0.05 vs. EGG group.

We first attempted to identify the effect of EGG itself on muscle signaling (Figure 2) and blood parameters (Figure 3) 1 h after the feeding. In response to each protein feeding, phosphorylation levels of Akt (Figure 2A), mTOR (Figure 2B), and S6 (Figure 2C) were amplified by several magnitudes compared to basal (being fasted) conditions. However, EGG-induced amplification was significantly lower for mTOR when compared to WHE (*p* < 0.05), and also weaker than both CAS and WHE in S6 (*p* < 0.05). For blood parameters, no significant difference was observed among groups except insulin (Figure 3B) where WHE feeding led to a higher increase compared to EGG feeding (*p* < 0.05).

### 3.2. Experiment II

**Figure 4:**
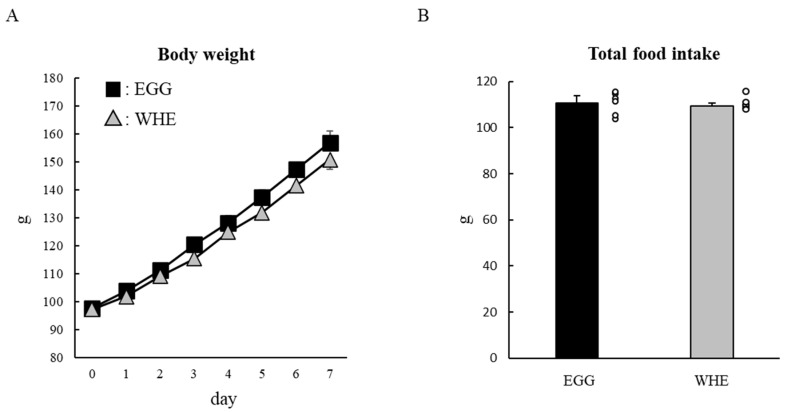
Chronic effect of each protein feeding on body weight and food intake (during 7 days). Rats were assigned to either an egg white, or whey protein diet group, and were maintained under ad libitum feeding for 7 days without clenbuterol administration. Bodyweight (**A**) and food intake were measured daily. Total food intake (**B**) was the sum of consumed food during 7 days. The dots beside bars indicate individual value. Values are means ± SE (*n* = 5). EGG: egg white protein, WHE: whey protein. Different superscript represents statistical differences among groups.

**Figure 5:**
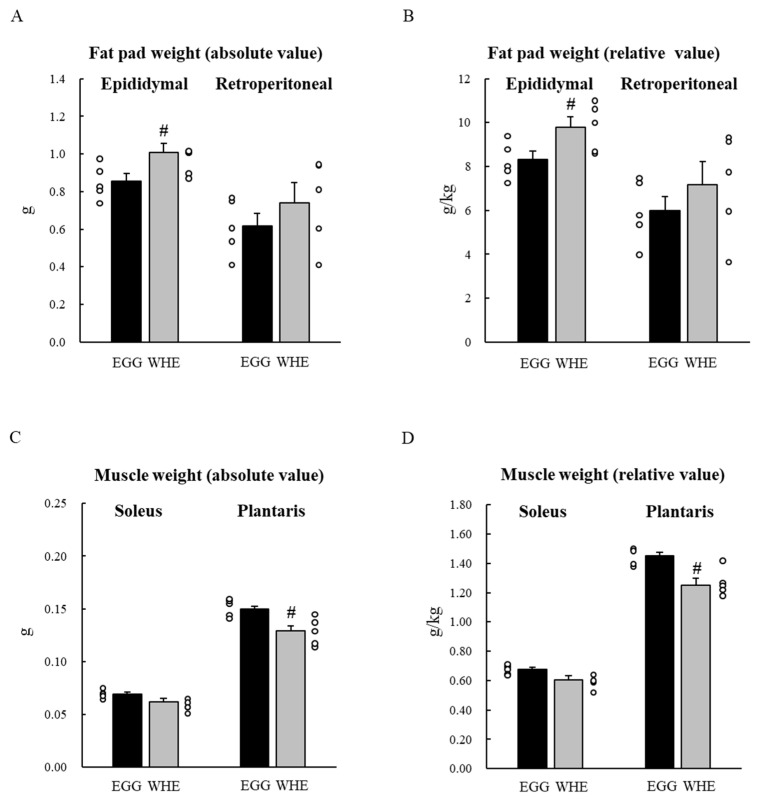
Chronic effect of each protein feeding on fat pad and muscle weight (Day 7). Rats were assigned to either an egg white, or whey protein diet group, and were maintained under ad libitum feeding for 7 days without clenbuterol administration. On day 7, rats were fasted for 6 h, and then fat pads (**A**,**B**) and muscles (**C**,**D**) were dissected out. An absolute value (**A**,**C**) and a relative value to body weight (**B**,**D**) were calculated. The dots beside bars indicate individual value. Values are means ± SE (*n* = 7). CAS: casein protein, EGG: egg white protein, WHE: whey protein. #: *p* < 0.05 vs. EGG group.

**Figure 6:**
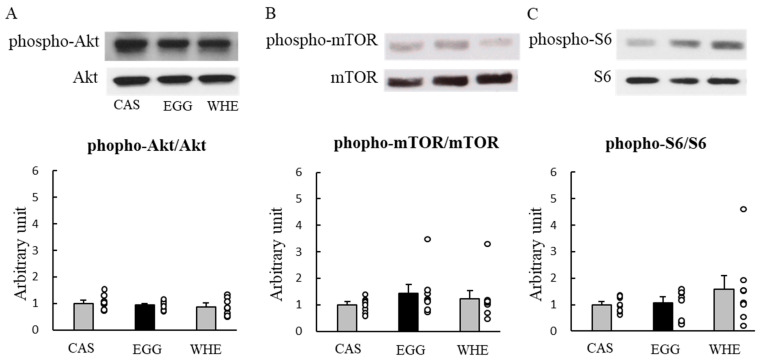
Chronic effect of each protein feeding on muscle signal transduction (Day 3). Rats were assigned to either a casein, egg white, or whey protein diet group, and were maintained under ad libitum feeding for 2 days without clenbuterol administration. On day 3, rats were fasted for 6 h, and then plantaris muscles were dissected out for western blot analysis of Akt (**A**), mTOR (**B**), or S6 (**C**). The dots beside bars indicate individual value. Values are means ± SE (*n* = 8). CAS: casein protein, EGG: egg white protein, WHE: whey protein.

Next, we examined the chronic effect of EGG feeding without clenbuterol treatment. Bodyweight throughout the experimental period was slightly higher following EGG feeding compared to WHE feeding, but it was not statistically significant (Figure 4A). Although total food intake was identical among the groups (Figure 4B), EGG feeding resulted in a significant and non-significant reduction in epididymal fat pad weight and retroperitoneal fat pad weight, respectively (Figure 5A,B; *p* < 0.05). Under these conditions, in plantaris muscle from rats fed EGG, we also found a greater muscle-gain effect (Figure 5C,D; *p* < 0.05). Regarding the soleus muscle, no difference was observed between groups. On day 3, when phosphorylation levels of Akt (Figure 6A), mTOR (Figure 6B), and S6 (Figure 6C) were measured after the 6-h fasting, these levels were comparable regardless of protein source.

### 3.3. Experiment III

**Figure 7:**
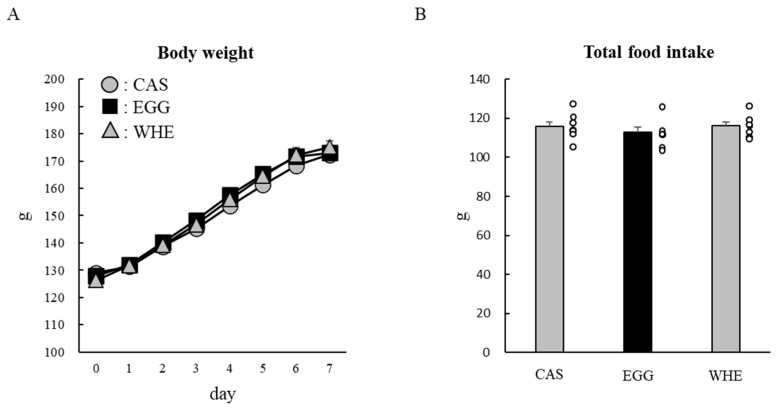
Chronic effect of each protein feeding with arginine-modification on body weight and food intake (during 7 days). Rats were assigned to either a casein, egg white, or whey protein diet group, and were maintained under ad libitum feeding for 7 days without clenbuterol administration. Bodyweight (**A**) and food intake were measured daily. Total food intake (**B**) was the sum of consumed food during 7 days. The dots beside bars indicate individual value. Values are means ± SE (*n* = 7). CAS: casein protein, EGG: egg white protein, WHE: whey protein.

**Figure 8:**
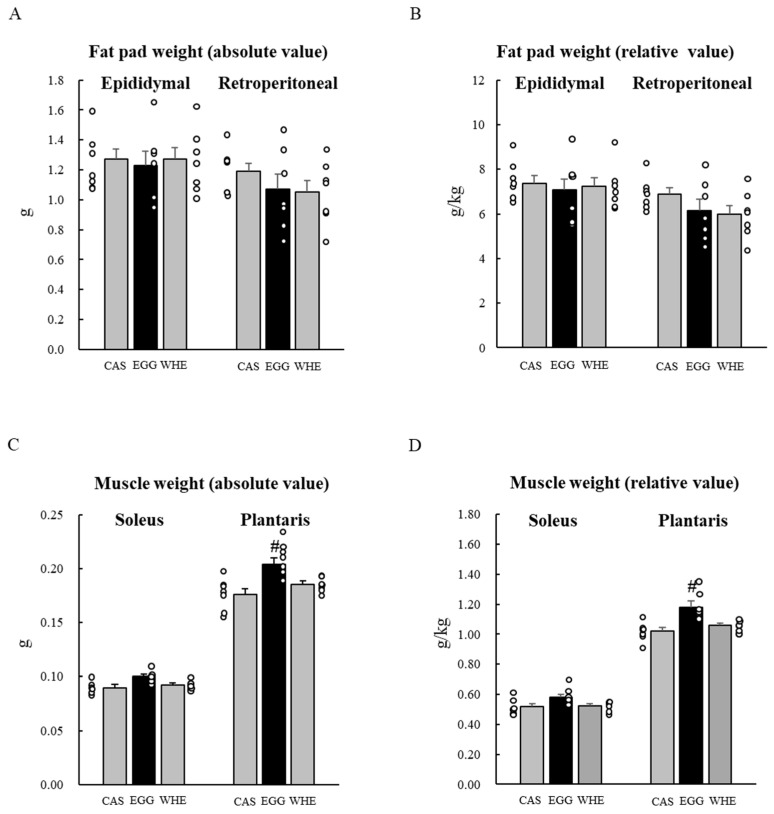
Chronic effect of each protein feeding with arginine-modification on fat pad and muscle weight (Day 7). Rats were assigned to either a casein, egg white, or whey protein diet group, and were maintained under ad libitum feeding for 7 days without clenbuterol administration. On day 7, rats were fasted for 6 h, and then fat pads (**A**,**B**) and muscles (**C**,**D**) were dissected out. An absolute value (**A**,**C**) and a relative value to body weight (**B**,**D**) were calculated. The dots beside bars indicate individual value. Values are means ± SE (*n* = 7). CAS: casein protein, EGG: egg white protein, WHE: whey protein. #: *p* < 0.05 vs. other two groups.

In this experiment, we analyzed amino acid compositions in each diet used thus far (left side of Table 2 as Diet 1). A typical characteristic of EGG was that the amount of branched-chain amino acids (leucine, isoleucine, and valine) was almost equivalent to that in CAS, and lower than in WHE. Whereas amounts of arginine were two-fold higher in EGG compared to other proteins. Arginine is known as amino acid functioning in anabolic response [21,22]. It has a visceral fat-lowering effect [23]. Therefore, to examine whether the stronger muscle-gain effect and the stronger visceral fat-lowering effect induced by EGG was due to the higher content of arginine in EGG, we prepared another diet named “arginine-modified” Diet 2 (right side of Table 2). During the 7-day feeding period with Diet 2, body weights were not different at any point (Figure 7A) and total food intake was identical among groups (Figure 7B). After the period, we could not find a significant difference in fad pad weight among groups (Figure 8A,B). In contrast, in skeletal muscle, the EGG-induced greater muscle-gain effect remained in plantaris muscle compared to other protein feedings (Figure 8C,D). The soleus muscle of EGG-fed rats was also relatively bigger than others, but it was not statistically different (Figure 8C,D).

### 3.4. Experiment IV

**Figure 9:**
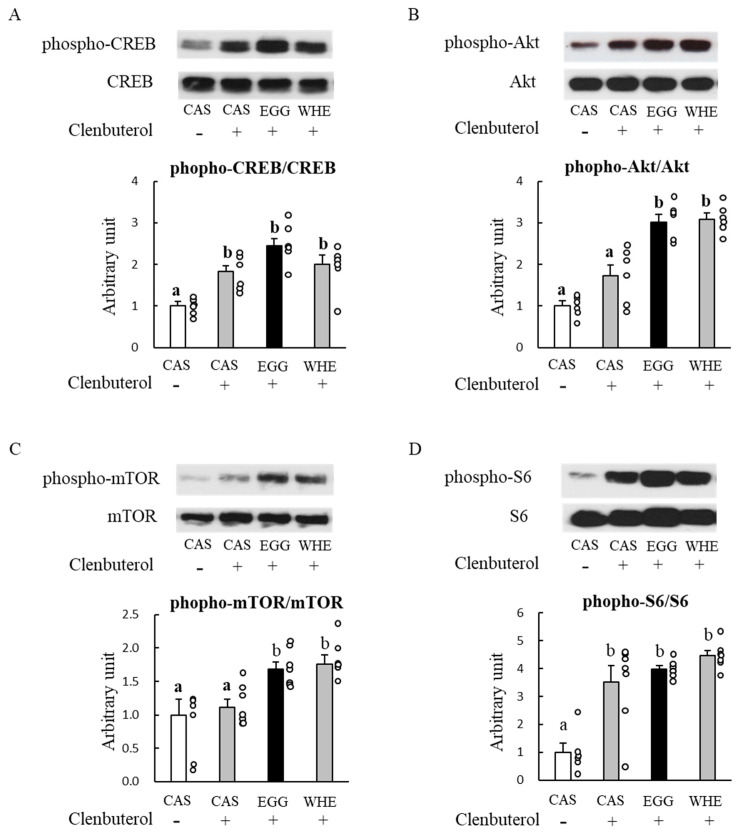
Acute effect of clenbuterol on muscle signal transduction (Day 3). Rats were assigned to either a casein, egg white, or whey protein diet group, and were maintained under ad libitum feeding for 2 days with/without saline or clenbuterol administration. On day 3, rats were administrated saline or clenbuterol. Two hours after the administration, plantaris muscles were dissected out for western blot analysis of CREB (**A**), Akt (**B**), mTOR (**C**), or S6 (**D**). The dots beside bars indicate individual value. Values are means ± SE (*n* = 6–7). CAS: casein protein, EGG: egg white protein, WHE: whey protein. Different superscript represents statistical differences among groups.

To examine whether the stronger muscle-gain effect of EGG was also superior to CAS and WHE under clenbuterol-treated conditions, we tested the acute effect of clenbuterol administration on these signaling molecules (Figure 9). Clenbuterol can increase cAMP level through activation of adenylate cyclase, and the increase finally leads to a CREB activation [24]. At 2 h post-administration, the CREB phosphorylation level, an indicator of cAMP-dependent response, was significantly amplified in all groups (A; *p* < 0.05 vs. CAS+saline) but the magnitude was identical among different protein sources. In these muscles, we also found considerable activation of Akt (B), mTOR (C), and S6 (D) in response to clenbuterol administration, with the lowest response observed in the CAS+clenbuterol group. In Akt and mTOR, these phosphorylation levels were significantly higher with EGG+clenbuterol and WHE+clenbuterol than those in the CAS+saline and CAS+clenbuterol groups (*p* < 0.05) but no significant difference was observed between EGG+clenbuterol and WHE+clenbuterol.

### 3.5. Experiment V

**Figure 10:**
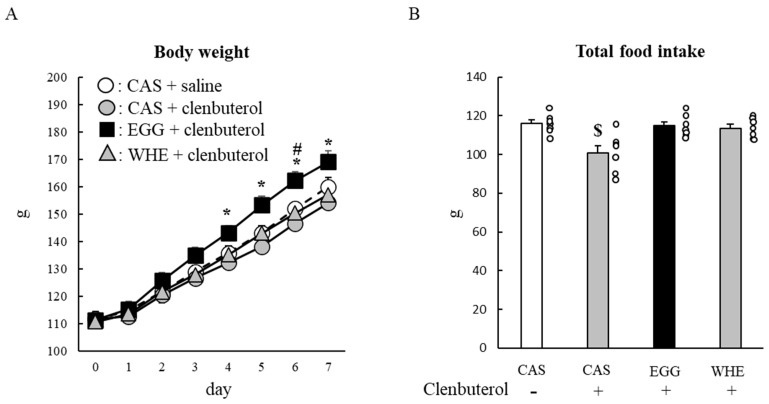
Chronic effect of each protein feeding on body weight and food intake (during 7 days). Rats were assigned to either a casein, egg white, or whey protein diet group, and were maintained under ad libitum feeding for 7 days with or without clenbuterol administration. Bodyweight (**A**) and food intake were measured daily. Total food intake (**B**) was the sum of consumed food during 7 days. The dots beside bars indicate individual value. Values are means ± SE (*n* = 7). CAS: casein protein, EGG: egg white protein, WHE: whey protein. *: *p* < 0.05 vs. CAS+clenbuterol group. #: *p* < 0.05 vs. WHE+clenbuterol group. $: *p* < 0.05 vs. other three groups.

We further examined the effect of chronic EGG feeding under clenbuterol treatment. During the 7-day period, body weights in all groups were gradually increased (Figure 10A). A tendency for greater gains began to appear in the EGG+clenbuterol group on day 2, and then on day 4, it reached a statistically significant level compared to the CAS+clenbuterol group (*p* < 0.05). Between EGG and WHE, body weight was greater in the EGG group throughout, but the difference was statistically significant only on day 6 (*p* < 0.05). The total food intake in the CAS+clenbuterol group was significantly lower than in other groups (Figure 10B; *p* < 0.05).

**Figure 11:**
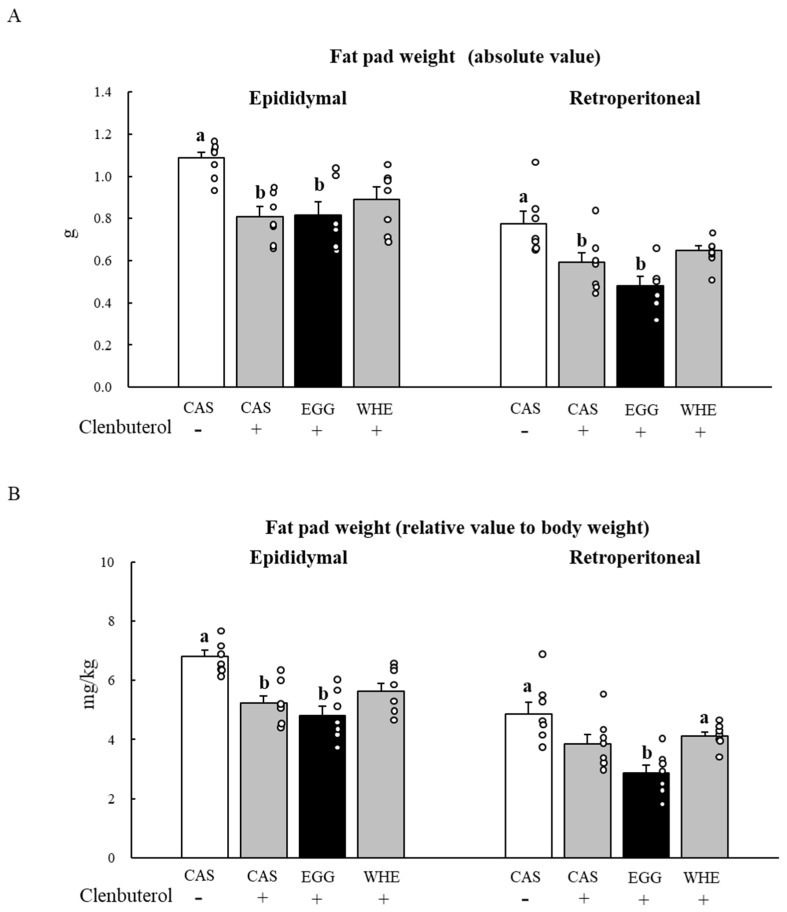
Chronic effect of each protein feeding on fat pad weight (Day 7). Rats were assigned to either a casein, egg white, or whey protein diet group, and were maintained under ad libitum feeding for 7 days with or without clenbuterol administration. On day 7, rats were fasted for 6 h, and then fat pads were dissected out. An absolute value (**A**) and a relative value to body weight (**B**) were calculated. The dots beside bars indicate individual value. Values are means ± SE (*n* = 7). CAS: casein protein, EGG: egg white protein, WHE: whey protein. Different superscript represents statistical difference among groups in same fad pad (*p* < 0.05).

In general, clenbuterol treatment decreased both epididymal and retroperitoneal fat pad weights (Figure 11). As shown, the greater body weight gain in the EGG group was not due to the facilitation of fat accumulation. Rather, fat pad weight after EGG feeding was the lowest compared to other groups, with a prominent decrease in the relative value of retroperitoneal fat pad weight when it was compared to the CAS+saline and WHE+clenbuterol groups (Figure 11B; *p* < 0.05).

**Figure 12:**
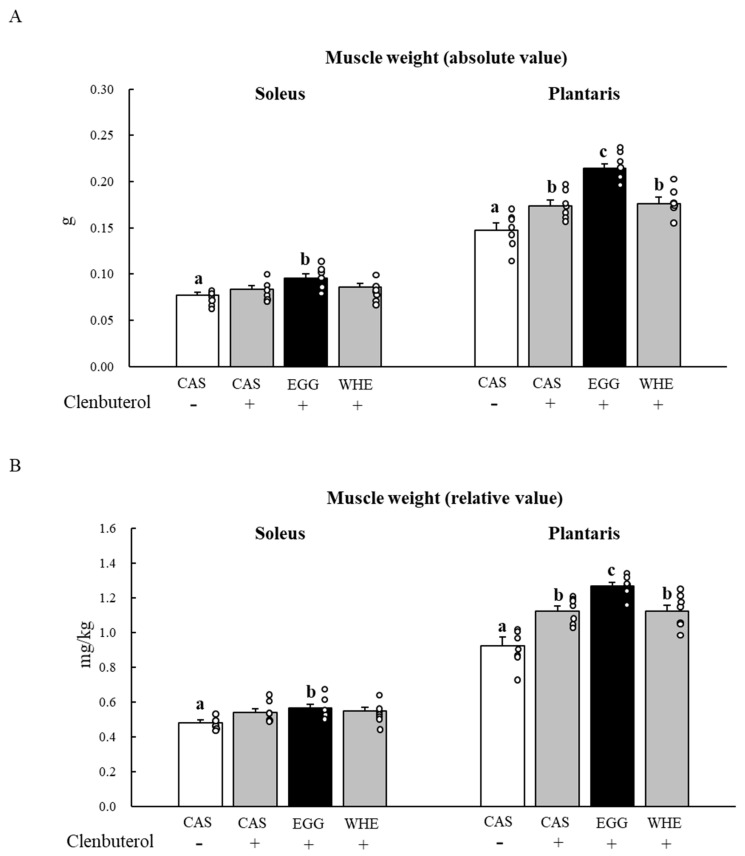
Chronic effect of each protein feeding on muscle weight (Day 7). Rats were assigned to either a casein, egg white, or whey protein diet group, and were maintained under ad libitum feeding for 7 days with or without clenbuterol administration. On day 7, rats were fasted for 6 h, and then muscles were dissected out. An absolute value (**A**) and a relative value to body weight (**B**) were calculated. The dots beside bars indicate individual value. Values are means ± SE (*n* = 7). CAS: casein protein, EGG: egg white protein, WHE: whey protein. Different superscript represents statistical difference among groups in same muscle (*p* < 0.05).

In contrast to the effect of EGG feeding on fat pad weight, a marked increase was observed in muscle gain (Figure 12). Clenbuterol treatment increased plantaris muscle weight in all groups, but the increase was significantly greater in the EGG group compared to other all groups (Figure 12A,B; *p* < 0.05). The same tendency was also observed in the soleus muscle (Figure 12A,B). The magnitude of the clenbuterol effect on the soleus muscle was relatively lower than that of the plantaris muscle but only EGG feeding led to a higher muscle gain compared to CAS+saline treatment (*p* < 0.05).

**Figure 13:**
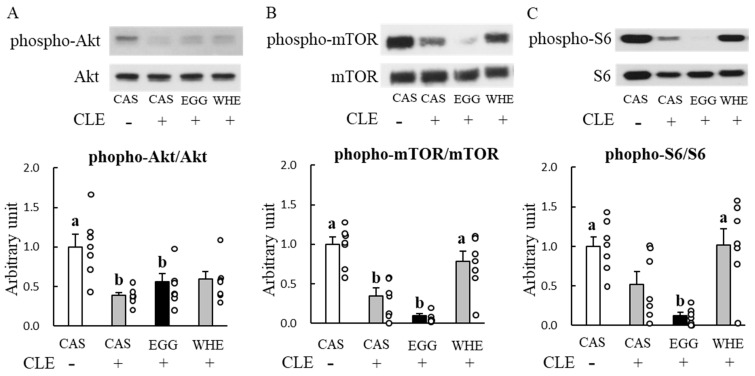
Chronic effect of each protein feeding on muscle signal transduction (Day 7). Rats were assigned to either a casein, egg white, or whey protein diet group, and were maintained under ad libitum feeding for 6 days with or without clenbuterol administration. On day 7, rats were fated for 6 h, and then plantaris muscles were dissected out for western blot analysis of Akt (**A**), mTOR (**B**), or S6 (**C**). The dots beside bars indicate individual value. Values are means ± SE (*n* = 7). CAS: casein protein, EGG: egg white protein, WHE: whey protein. CLE: clenbuterol. Different superscript represents statistical differences among groups.

To determine the molecular mechanism behind the EGG-induced facilitation in muscle gain, we examined the effect of EGG feeding on the phosphorylation levels of Akt and mTOR signaling molecules (Figure 13). Although we expected to see an increase following chronic (7-day) clenbuterol treatment in the phosphorylation state of Akt (A) and mTOR (B), we observed a marked downregulation of these phosphorylation levels after 6-h fasting and 20 h post-clenbuterol administration, especially in the case of EGG feeding. The same tendency was also observed in phospho-S6 (C) which is a downstream target of mTOR. The levels of mTOR and S6 in the EGG+clenbuterol group were significantly lower than those in the CAS+saline and WHE+clenbuterol (*p* < 0.05) groups when these were measured 20 h after the last clenbuterol administration and the 6-h fasting.

## 4. Discussion

Aside from the negative concept linking egg consumption and the adverse effects of cholesterol, there is accumulating evidence that eggs and egg-derived foods perform positive functions in promoting health [25]. In regards to consumption of EGG and EGG hydrolysate, several previous studies conducted by Ochiai et al. have provided evidence that these protein feedings inhibited the accumulation of intracellular triglyceride in the liver and skeletal muscle together with visceral mass in insulin-resistant animals compared to those consuming CAS [17,18,19]. They also found EGG to have an inhibitory effect on food intake when fed a high-fat diet, and they expanded their measurement of this to include muscle gain. One study [18] demonstrated that EGG feeding induced greater muscle gain despite lower food intake, and another study [19] showed that this effect of EGG on muscle was not observed when it was compared under pair-fed conditions. These results seem difficult to interpret for us, and we did not observe the inhibitory effect on food intake; however, we identified the stronger visceral fat-reducing effect in the EGG group compared to both the CAS and WHE groups. Note that in these studies muscle mass was expressed as a value relative to body weight [17,18,19], so bodyweight reduction accompanied by a visceral fat mass reduction in EGG feeding might influence the muscle mass calculation through a secondary effect [18]. In the present study, we clearly demonstrated that the main result of EGG feeding is muscle gain, in the calculation of both absolute and relative value, under anabolism-dominant conditions. This was demonstrated in comparison to not only CAS but also WHE, and regardless of a lower leucine amount.

We also provided a result showing that EGG-induced facilitation in muscle gain is apparent without clenbuterol treatment. Therefore, we focused on the contribution of EGG itself. When EGG was ingested, activation levels of mTOR and S6 were statistically lower than those of other protein sources or WHE 1 h after EGG feeding. Although we did not measure leucine concentration in blood, the level might be lower in EGG than others, and this would be a plausible candidate for lower activation of mTOR signaling. Also, given that insulin is capable of activating mTOR [9], lower insulin secretion response after EGG would affect mTOR activation levels observed 1 h after EGG feeding.

Although EGG has an almost equivalent or lower leucine amount than CAS and WHE, it is known to be an arginine-rich protein source [15]. This higher arginine amount in EGG was one of the plausible explanations for the mechanism of its stronger muscle-gain effect on skeletal muscle, as arginine stimulates the anabolic hormone secretion. For example, a single arginine administration stimulated insulin [21] and growth hormone [22] secretion immediately after administration. In the present study, blood parameter responses immediately after each protein feeding were similar among groups except for insulin (Figure 10), which was lower in rats fed EGG. We cannot exclude the possibility that an insufficient number of blood sampling points might confound the overall trend. However, under our limited experimental conditions, the acute response of IGF-1, GH, and insulin could not explain the stronger muscle-gain effect of EGG.

Whereas young mammals have been used as a good experimental model to estimate the effect of nutrients or food on the whole body or fractional anabolic effect, it should be noted that arginine is categorized as a semi-essential amino acid and is needed for optimal growth in young mammals, but not in adults [26]. This is also true in rats and is supported by a study by Milner et al. [27], which demonstrated that rats fed an arginine-deficient diet, from immediately post-lactation until 18 days later, showed lower body weights compared to those fed a standard diet. In contrast to the results in the comparison to definitive conditions like “deficient”, Suzumura et al. [28] demonstrated that when young (5-weeks old) rats ate a 20% CAS-based diet with additional arginine supplementation for 10 days, body weights were not different from the 20% CAS-based diet without additional arginine supplementation. This result implies that even during a growing period, arginine demands in the body and arginine supply (arginine intake and its synthesis) might reach a balancing point for skeletal muscle when rats eat arginine presented in 20% CAS. Besides these surrounding evidence for our interpretation, we could not find direct evidence showing the arginine-independent effect of EGG feeding in the previous study. Definitely, by using an arginine-modified diet, we again confirmed the biggest muscle mass in rats fed EGG diet. This is the first evidence, to our knowledge, to demonstrate that EGG-induced facilitation in muscle gain is not dependent on the arginine content in the protein source.

We found that EGG feeding resulted in a marked reduction in visceral fat mass under growth and clenbuterol-treated conditions. This is in accordance with previous studies [17,18,19] but the mechanism has not been well documented. Regarding this question, we found that an arginine-modified diet eliminated the fat-reducing effect of the EGG. Previously, Fu et al. [23] demonstrated that additional arginine supplementation for 10 weeks reduced visceral fat mass with marked bodyweight reduction in Zucker diabetic fatty rats, through arginine-dependent nitric oxide production. In that study, the authors also found that skeletal muscle mass was increased after additional arginine supplementation when it was expressed as a relative value, whereas it was identical when it was expressed as an absolute value. Given these results, our present results indicate that in contrast to the case of skeletal muscle, the abundant arginine content of EGG plays a predominant role in facilitating visceral fat mass reduction after EGG feeding.

To emphasize the potential of EGG and to see whether the EGG-induced facilitation in muscle gain would appear under highly anabolism-dominant conditions, we used clenbuterol. Clenbuterol has been known to be a strong anabolic reagent and it can induce significant muscle hypertrophy within a few days [29,30,31]. In accordance with these results, we successfully produced muscle hypertrophy after a 7-day treatment with the 1 mg/kg clenbuterol. This was observed mainly in fast-twitch glycolytic plantaris muscle, regardless of protein source. Traditionally, it has been thought that clenbuterol induces hypertrophy through a pathway related to adenylate cyclase-mediated cAMP production and the subsequent protein kinase A-CREB activation pathway [24]. More recently, in a manner similar to the leucine-induced anabolic effect, activation of the phosphatidylinositol 3-kinase (PI3K)-Akt-mTOR pathway has been recognized to regulate the clenbuterol-induced anabolic effect [32]. When we measured phosphorylation levels of Akt and mTOR at day 7, following a 6-h fasting and 20 h post-clenbuterol administration, phospho-Akt and phospho-mTOR levels were downregulated in all groups treated with clenbuterol (Figure 13), and the lowest these levels were observed in the most hypertrophied muscle in rats fed EGG. Because we did not measure blood parameters in this situation, we cannot mention whether the changes in blood would affect muscle signaling. However, our signaling results are not surprising because generally most, but not all [32], clenbuterol-induced anabolic responses (e.g., activated anabolic signaling and muscle protein synthesis) were more pronounced 2 to 3 days after starting clenbuterol treatment, and returned to basal or lower than basal levels after 7 days or more [31], although the increased muscle mass persisted [30]. We interpret these downregulations as proof of the most powerful effect of EGG in terms of negative feedback for preventing excess hypertrophy. Concurrently, these results also could suggest that the stronger muscle-gain effect of EGG is unlikely to be due to the absence of adequate negative feedback regulation. To further investigate, we also examined the acute effect of clenbuterol on these phosphorylations as well as CREB levels 2 h after injection. Although those in the CAS group were relatively lower compared to other protein sources, the highest increase in these activations was not observed in rats fed EGG. Taken together, these results indicate that sensitivity against clenbuterol to mTOR and insulin signaling is unlikely to explain the mechanism of EGG-induced facilitation in muscle gain.

We have also provided insight into clenbuterol-induced anabolic effects on skeletal muscle. Clenbuterol has a powerful anabolic effect and appears to cause physiological skeletal mass to achieve maximum efficiency. However, few animal studies have shown clenbuterol treatment to result in an additive anabolic response when combined with muscle activity-associated stimuli/conditions (i.e., tenotomy-induced compensatory hypertrophy) [29] or recovery conditions from immobilization-induced atrophy [33]. Similarly, GH administration was also reported to amplify the anabolic effect of clenbuterol [34]. To the best of our knowledge, this is the first evidence that the protein source, especially EGG, is an additional factor for accelerating the anabolic efficiency of clenbuterol. It is known that clenbuterol is a beta-adrenergic agonist and the potential contribution of the beta-adrenergic pathway for exercise-induced hypertrophy has been documented [35]. EGG might be a beneficial protein source for athletes with a desire to increase their muscle mass.

In the present study, we had only single sampling points for blood and muscle signal transductions, therefore this is a limitation and a point for further discussion. Nevertheless, our effort to identify the mechanism for the stronger muscle-gain effect induced by EGG on mTOR and insulin signaling might not be a good strategy considering our experimental conditions. Insufficient activation of mTOR signaling after single-dose EGG ingestion has also been reported previously [36]. Additional examination of relevant signaling pathways including muscle protein breakdown pathways would provide a clearer interpretation. Alternatively, whole egg, egg-derived foods including EGG, and egg-derived peptides are gaining attention because of their reported functions in countering type 2 diabetes via their antioxidant and anti-inflammatory properties [37]. The mechanism which is attributed to one or more common factors related to these positive effects might have some role in the present stronger muscle-gain effect in EGG. Further study is needed to resolve this molecular mechanism.

As another limitation of our experiments, we did not measure the effect of EGG feeding on muscle power output, muscle fiber-type compositions, or cross-sectional area. These surrounding results would be convincing proof. In addition, we cannot conclude from the present study whether EGG can induce its stronger muscle-gain effect under conditions that are catabolism-dominant, such as cachexia, malnutrition-associated muscle atrophy, muscle disuse, or sarcopenia associated with these conditions. We hope that these questions can be answered by future research.

## 5. Conclusions

In the present study, we demonstrated that EGG facilitates skeletal muscle gain under highly anabolism-dominant conditions compared to CAS and WHE and this stronger muscle-gain effect of EGG is not dependent on an arginine-rich protein source. The plausible mechanism of the stronger muscle-gain effect induced by EGG was not detectable in mTOR and insulin signaling under our experimental conditions. We conclude that EGG may have a superior efficiency in muscle gain compared to other common animal-based proteins.

## Figures and Tables

**Table 1:** Composition of nutrients in protein sources and diets.

	CAS	EGG	WHE
**In each protein source (g/100g)**			
Protein	91	81	78
Fat	1	1	6
**In each diet (Diet 1) (g/100g)**			
Casein protein	22	-	-
Egg white protein	-	25	-
Whey protein	-	-	26
Sucrose	5	5	5
α-Corn starch	46	47	47
Corn oil	11	7	6
Vitamin mix (AIN-76)	2	2	2
Mineral mix (AIN-76)	6	6	6
Cellulose	8	8	8
Final protein content	20	20	20
Final fat content	12	12	12

**Table 2:** Composition of amino acid.

	Original (Diet 1)			Arginine-modified (Diet 2)		
(mg/100g)	CAS	EGG	WHE	CAS	EGG	WHE
Arginine	610	1070	438	1063	1063	1063
Lysine	1400	1290	1790	1391	1282	1749
Histidine	457	385	332	454	383	330
Phenylalanine	935	1130	605	929	1123	601
Tyrosine	924	722	596	918	717	592
Leucine	1760	1600	2210	1749	1590	2196
Isoleucine	611	643	957	607	639	951
Methionine	554	750	452	551	745	449
Valine	788	844	877	783	839	871
Alanine	616	1240	1120	612	1232	1113
Glycine	357	730	383	355	725	381
Proline	2080	714	1280	2238	1338	1272
Glutamic acid	4370	2720	3770	4343	2703	3746
Serine	1120	1500	1160	1113	1491	1153
Threonine	764	907	1380	759	901	1371
Aspartic acid	1410	2150	2290	1401	2136	2276
Tryptophan	228	296	367	227	294	365
Cysteine	87	598	508	86	594	505

## Data Availability

Each plot is in the article.

## References

[1] Atherton P.J., Smith K. (2012). Muscle protein synthesis in response to nutrition and exercise. J. Physiol..

[2] Jäger R., Kerksick C.M., Campbell B.I., Cribb P.J., Wells S.D., Skwiat T.M., Purpura M., Ziegenfuss T.N., Ferrando A.A., Arent S.M. (2017). International Society of Sports Nutrition Position Stand: Protein and exercise. J. Int. Soc. Sports Nutr..

[3] Putra C., Konow N., Gage M., York C.G., Mangano K.M. (2021). Protein source and muscle health in older adults: A literature review. Nutrients.

[4] Hoffman J.R., Falvo M.J. (2004). Protein—Which is best?. J. Sports Sci. Med..

[5] Mathai J.K., Liu Y., Stein H.H. (2017). Values for digestible indispensable amino acid scores (DIAAS) for some dairy and plant proteins may better describe protein quality than values calculated using the concept for protein digestibility-corrected amino acid scores (PDCAAS). Br. J. Nutr..

[6] Tang J.E., Moore D.R., Kujbida G.W., Tamopolsky M.A., Phillips S.M. (2009). Ingestion of whey hydrolysate, casein, or soy protein isolate: Effects on mixed muscle protein synthesis at rest and following resistance exercise in young man. J. Appl. Physiol..

[7] Yang Y., Churchward-Venne T.A., Burd N.A., Breen L., Tarnopolsky M.A., Phillips S.M. (2012). Myofibrillar protein synthesis following ingestion of soy protein isolate at rest and after resistance exercise in elderly man. Nutr. Metab..

[8] Volek J.S., Volk B.M., Gómez A.L., Kunces L.J., Kupchak B.R., Freidenreich D.J., Aristizabal J.C., Saenz C., Dunn-Lewis C., Ballard K.D. (2013). Whey protein supplementation during resistance training augments lean body mass. J. Am. Coll. Nutr..

[9] Ham D.J., Caldow M.K., Lynch G.S., Koopman R. (2014). Leucine as a treatment for muscle wasting: A critical review. Clin. Nutr..

[10] Crozier S.J., Kimball S.R., Emmert S.W., Anthony J.C., Jefferson L.S. (2005). Oral leucine administration stimulates protein synthesis in rat skeletal muscle. J. Nutr..

[11] Wilkinson D.J., Hossain T., Hill D.S., Phillips B.E., Crossland H., Williams J., Loughna P., Churchward-Venne T.A., Breen L., Phillips S.M. (2013). Effects of leucine and its metabolite β-hydroxy-β-methylbutyrate on human skeletal muscle protein metabolism. J. Physiol..

[12] Churchward-Venne T.A., Burd N.A., Mitchell C.J., West D.W.D., Philp A., Marcotte G.R., Baker S.K., Baar K., Phillips S.M. (2012). Supplementation of a suboptimal protein dose with leucine or essential amino acids: Effects on myofibrillar protein synthesis at rest and following resistance exercise in men. J. Physiol..

[13] Devries M.C., McGlory C., Bolster D.R., Kamil A., Rahn M., Harkness L., Baker S.K., Phillips S.M. (2018). Leucine, not total protein, content of a supplement is the primary determinant of muscle protein anabolic responses in healthy older women. J. Nutr..

[14] Gorissen S.H.M., Crombag J.J.R., Senden J.M.G., Waterval W.A.H., Bierau J., Verdjik L.B., van Loon L.J.C. (2018). Protein content and amino acid composition of commercially available plant-based protein isolates. Amino Acid.

[15] Matsuoka R., Kurihara H., Nishijima N., Oda Y., Handa A. (2019). Egg White hydrolysate retains the nutritional value of proteins and is quickly absorbed in Rats. Sci. World J..

[16] Kato Y., Sawada A., Numao S., Suzuki M. (2011). chronic effect of light resistance exercise after ingestion of a high-protein snack on increase of skeletal muscle mass and strength in young adults. J. Nutr. Sci. Vitaminol..

[17] Ochiai M., Kuroda T., Matsuo T. (2014). Increased muscular triglyceride content and hyperglycemia in Goto-Kakizaki rat are decreased by egg white hydrolysate. Int. J. Food Sci. Nutr..

[18] Ochiai M., Matsuo T. (2014). Effect of egg white and its hydrolysate on stearoyl-CoA desaturase index and fat accumulation in rat tissues. Int. J. Food Sci. Nutr..

[19] Ochiai M., Misaki K., Takeuchi T., Narumi R., Azuma Y., Matsuo T. (2017). Egg White hydrolysate can be a low-allergenic food material to suppress ectopic fat accumulation in rats fed an equicaloric diet. J. Nutr. Sci. Vitaminol..

[20] Matsuoka R., Takahashi Y., Kimura M., Masuda Y., Kunou M. (2017). Heating has no effect on the net protein utilization from egg whites in rats. Sci. World J..

[21] Parmer J.P., Benson J.W., Walter R.M., Ensinck J.W. (1976). Arginine-stimulated acute phase of insulin and glucagon secretion in diabetic subjects. J. Clin. Investig..

[22] Alba-Roth J., Müller O.A., Schopohl J., von Werder K. (1988). Arginine stimulates growth hormone secretion by suppressing endogenous somatostatin secretion. J. Clin. Endocrinol. Metab..

[23] Fu W.J., Haynes T.E., Kohli R., Hu J., Shi W., Spencer T.E., Carroll R.J., Meininger C.J., Wu G. (2005). Dietary L-arginine supplementation reduces fat mass in Zucker diabetic fatty rats. J. Nutr..

[24] Kitaura T. (2013). How β2-adrenergic agonists induce skeletal muscle hypertrophy?. J. Phys. Fitness Sports Med..

[25] Miranda J.M., Anton X., Redondo-Valbuena C., Roca-Saavedra P., Rodriguez J.A., Lamas A., Franco C.M., Cepeda A. (2015). Egg and egg-derived foods: Effects on human health and use as functional foods. Nutrients.

[26] Tapiero H., Mathé G., Couvreur P., Tew K.D. (2002). I. Arginine. Biomed. Pharmacother..

[27] Milner J.A., Wakeling A.E., Visek W.J. (1974). Effect of arginine deficiency on growth and intermediary metabolism in rats. J. Nutr..

[28] Suzumura S., Tujioka K., Yamada T., Yokogoshi H., Akizuki S., Hishida Y., Tsutsui K., Hayase K. (2015). Comparison of the Effects of Ornithine and Arginine on the Brain Protein Synthesis Rate in Young Rats. J. Nutr. Sci. Vitam..

[29] Maltin C.A., Delday M.L., Hay S.M., Smith F.G., Lobley G.E., Reeds P.J. (1987). The effect of the anabolic agent, clenbuterol, on overloaded rat skeletal muscle. Biosci. Rep..

[30] Maltin C.A., Hay S.M., McMillan D.N., Delday M.I. (1992). Tissue specific responses to clenbuterol; temporal changes in protein metabolism of striated muscle and visceral tissues from rats. Growth Regul..

[31] Sneddon A.A., Delday M.I., Steven J., Maltin C.A. (2001). Elevated IGF-II mRNA and phosphorylation of 4E-BP1 and p70S6k in muscle showing clenbuterol-induced anabolism. Am. J. Physiol. Endocrinol. Metab..

[32] Kline W.O., Panaro F.J., Yang H., Bodine S.C. (2007). Rapamycin inhibits the growth and muscle-sparing effects of clenbuterol. J. Appl. Physiol..

[33] Suzuki H., Yoshikawa Y., Tsujimoto H., Kitaura T., Muraoka I. (2020). Clenbuterol accelerates recovery after immobilization-induced atrophy of rat hindlimb muscle. Acta Histochem..

[34] Maltin C.A., Delday M.I., Hay S.M., Innes G.M., Williams P.E. (1990). Effects of bovine pituitary growth hormone alone or in combination with the beta-agonist clenbuterol on muscle growth and composition in veal calves. Br. J. Nutr..

[35] Berdeaux R., Stewart R. (2012). cAMP signaling in skeletal muscle adaptation: Hypertrophy, metabolism, and regeneration. Am. J. Physiol. Endocrinol. Metab..

[36] Moore D.R., Robinson M.J., Fry J.L., Tang J.E., Glover E.I., Wilkinson S.B., Prior T., Tarnopolsky M.A., Phillips S.M. (2009). Ingested protein dose response of muscle and albumin protein synthesis after resistance exercise in young men. Am. J. Clin. Nutr..

[37] Wang X., Son M., Meram C.D., Wu J. (2019). Mechanism and potential of egg consumption and egg bioactive components on type-2 diabetes. Nutrients.

